# Oral Rehabilitation of a Patient With Generalized Inflammatory Gingival Overgrowth Exacerbated by Felodipine: A Case Report

**DOI:** 10.7759/cureus.24332

**Published:** 2022-04-21

**Authors:** Wan Ahmad Faiz Wan Jamil, Matheel AL-Rawas, Huwaina Abd Ghani, Rabihah Alawi, Yanti Johari

**Affiliations:** 1 Unit of Restorative Dentistry, Dental Health District Office of Kuala Krai, Kelantan, MYS; 2 Prosthodontic Unit, Hospital Universiti Sains Malaysia, Kelantan, MYS; 3 Prosthodontic Unit, School of Dental Sciences, Health Campus, Universiti Sains Malaysia, Kelantan, MYS; 4 Conservative Dentistry Unit, School of Dental Sciences, Health Campus, Universiti Sains Malaysia, Kelantan, MYS

**Keywords:** fixed prosthodontics, sanitary pontic, hygienic pontic, calcium channel blockers, felodipine, gingival hyperplasia, gingival overgrowth

## Abstract

Gingival enlargement may manifest as a side effect of medications (calcium channel blockers, anticonvulsants, or immunosuppressants) and may be associated with inflammation, malignancy, or genetic inheritance. This condition has a significant impact on a patient’s quality of life and affects their oral health status. This case report describes the management of a 68-year-old gentleman who presented with generalized gingival enlargement and chronic periapical abscess originating from tooth 34, which served as an abutment for a fixed partial prosthesis. The patient's medical history revealed that felodipine, an antihypertensive medication, was prescribed to him. A comprehensive treatment plan was developed to improve the patient’s quality of life.

## Introduction

Gingival enlargement (GE) is clinically presented as enlarged interdental papillae with lobulated morphology, bleeding upon probing, and a compromised appearance of the tooth. In some severe cases, it may affect function, namely speech and mastication, as well as aesthetics. Poor oral hygiene, genetic factors, individual susceptibility, and drug interaction with fibroblasts were all factors that contributed to the occurrence of GE [1]. Chronic inflammatory changes are common in cases of GE due to prolonged chronic irritation of marginal gingival tissue by either plaque or calculus formation or poorly contoured restoration or fitting of dental prostheses [2]. Certain systemic medications may lead to drug-influenced gingival enlargement (DIGE) [3], particularly anticonvulsants (phenytoin), immunosuppressors (cyclosporin), and antihypertensives [calcium channel blockers (CCB)]. Felodipine, the second generation of dihydropyridine (CCB), was first reported to cause DIGE in 1991 [4]. Compared to the first generation of CCB (nifedipine), there was less incidence of DIGE related to the second (felodipine) and third (amlodipine) generations of CCB [5]. The second class of CCBs was commonly used to treat a variety of cardiovascular diseases, including hypertension, angina pectoris, and cardiac arrhythmias, by acting on voltage-dependent calcium ion channels in smooth muscle and promoting vasodilation. Eventually, it may affect the concentration of calcium ions, both extracellular and intracellular, which in turn can influence the function of collagenase [6], hence indirectly playing a role in the initiation and progression of DIGE.

The development of DIGE often cannot be explained by the medication per se, as other factors need to be ruled out during the diagnostic process. Another factor that plays an important role in the pathophysiology of DIGE is the inflammatory reaction due to biofilm. Even though the mechanism of DIGE is not clear, the cellular inflammatory events may be altered by the presence of systemic medication, affecting the fibroblast activity and hence leading to the clinical sign of DIGE [7]. DIGE has also been reported to be associated with other factors, including diabetes. DIGE has been linked to uncontrolled type two diabetes mellitus in several case reports [8,9]. It was speculated that in hyperglycemia events, the formation of advanced glycation end-products (AGEs) that bind to specific receptors (RAGEs) might lead to an elevated local inflammatory response which eventually could cause DIGE [10]. This case report describes the management of a 68-year-old gentleman who presented with generalized gingival enlargement and chronic periapical abscess of tooth 34, which served as an abutment of a fixed partial prosthesis. Thus, a thorough clinical examination and multidisciplinary management involving a good rapport with the medical team were essential in managing this DIGE case. 

## Case presentation

**Figure 1:**
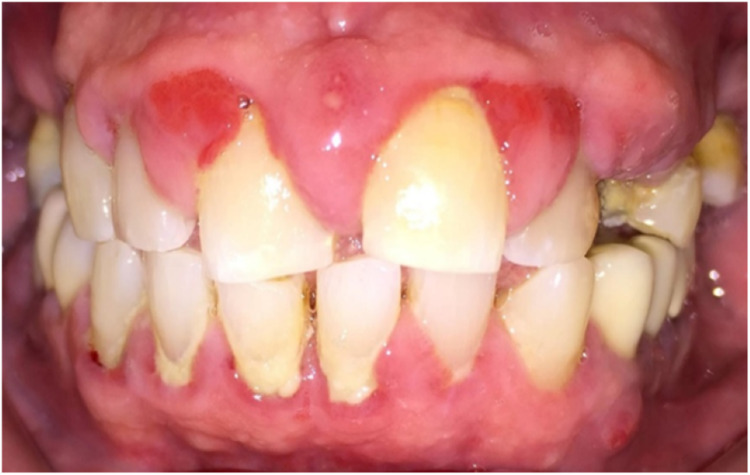
Clinical presentation at the initial visit.

**Figure 2:**
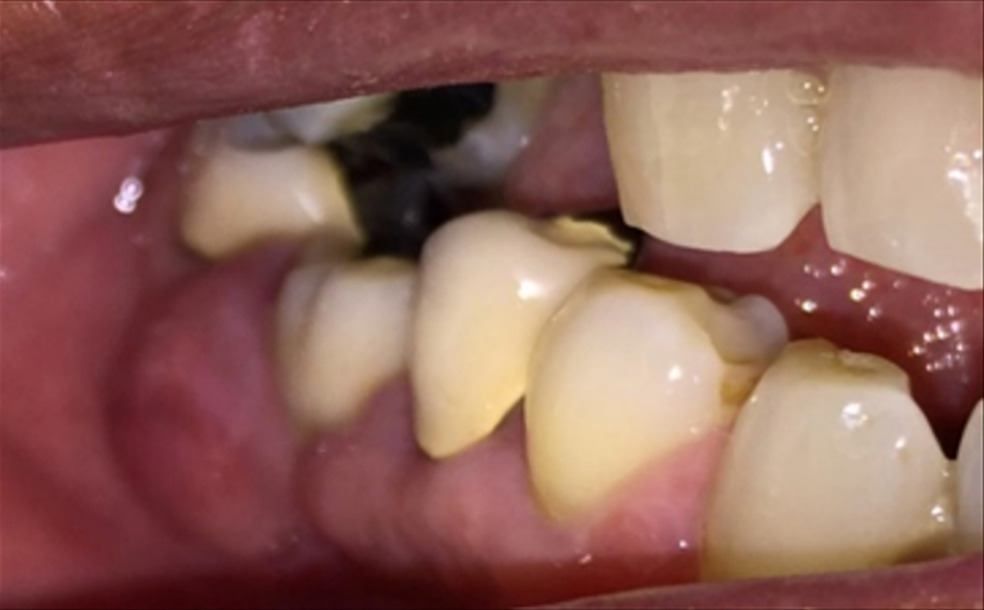
Gingival enlargement beneath the pontic of the bridge from teeth 45 to 47.

**Figure 3:**
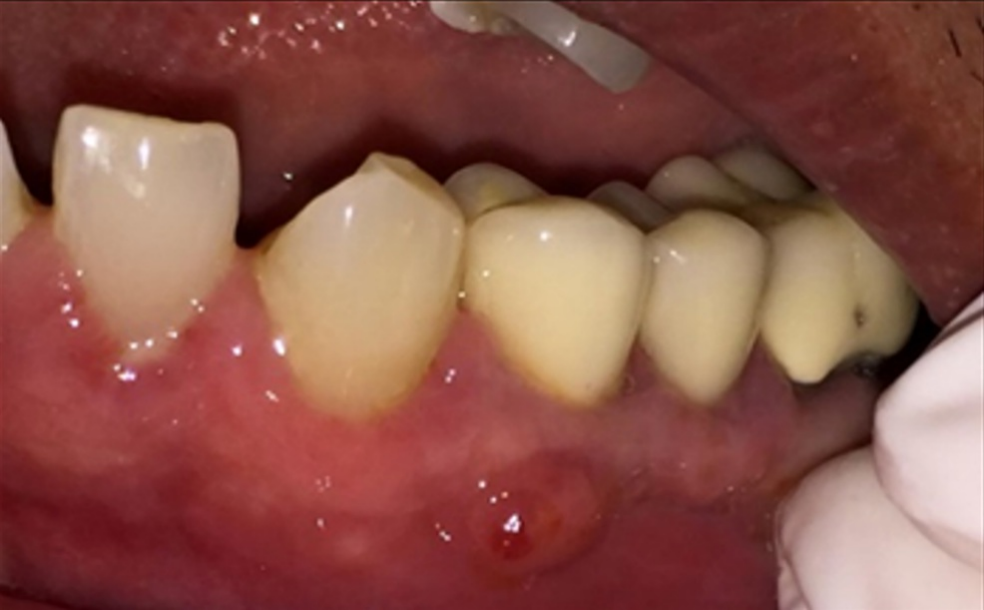
Sinus discharge from abutment tooth 34.

**Figure 4:**
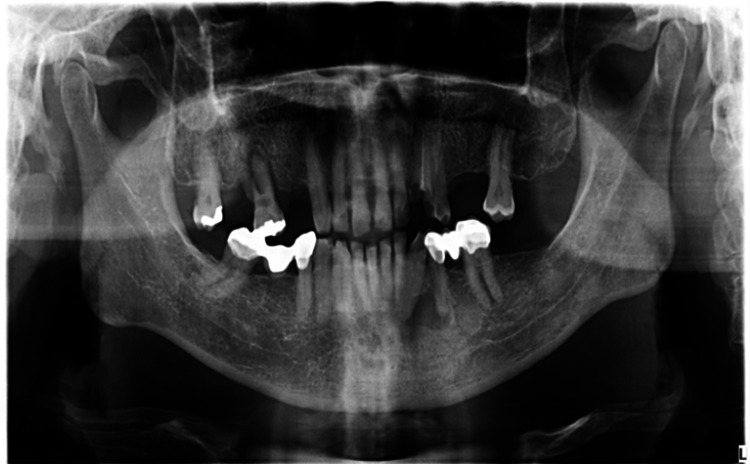
Panoramic radiograph of the patient.

A 68-year-old gentleman was presented with generalized gingival overgrowth and chronic periapical abscess of tooth 34. He complained about the persistent gingival swelling that has been present for the past three months, and that bleeds spontaneously while brushing. He also experienced a throbbing pain from the lower left posterior tooth, eventually resulting in pus discharge. Due to his medical condition, he has been on polypharmacy for diabetes mellitus type two (Glucovance® one tablet daily), hypercholesterolemia (Simvastatin 10 mg at night), and hypertension (Felodipine 10mg OD, Indapamide 1.5mg OD, Valsartan 80mg OD). The patient was a symptomatic dental attendee, and his last dental visit was in 2009 for a fixed prosthodontics treatment on the lower dental arch (three units fixed-fixed bridge of teeth 34 to 36, and teeth 45 to 47. Intraorally, there was generalized GE and inflammation plus abundant calculus with swollen interdental papillae, a fiery red, granular, pebbly surface that extended from marginal to attached gingiva, especially around the upper anterior teeth (Figure 1). There was also GE detected beneath the pontic of a poorly designed fixed bridge from tooth 45 to tooth 47 (Figure 2). This was made more complicated by the over erupted tooth 16 with grade II mobility and grade II furcation involvement along with palatal root caries. The multiple loss of upper posterior teeth was not restored with any prosthesis. There was sinus discharge associated with the abutment tooth 34 (Figure 3) and the presence of a retained root of 23, which appears to be beyond restorable. The patient’s oral hygiene was poor, with the basic periodontal examination (BPE) score 3, indicated by abundant plaque and calculus resulting from a lack of awareness of oral hygiene practices. The presence of pseudopockets of 3 to 7 mm depths as a result of GE was also noted. A panoramic radiograph showed extensive periodontal ligament space widening of tooth 16 while the retained root of 23 was root canal treated, which was a few millimeters short from the apex. There was unilocular radiolucency (size 11 mm X 9 mm) at the apical part of tooth 34, extending to tooth 33 (Figure 4).

**Figure 5:**
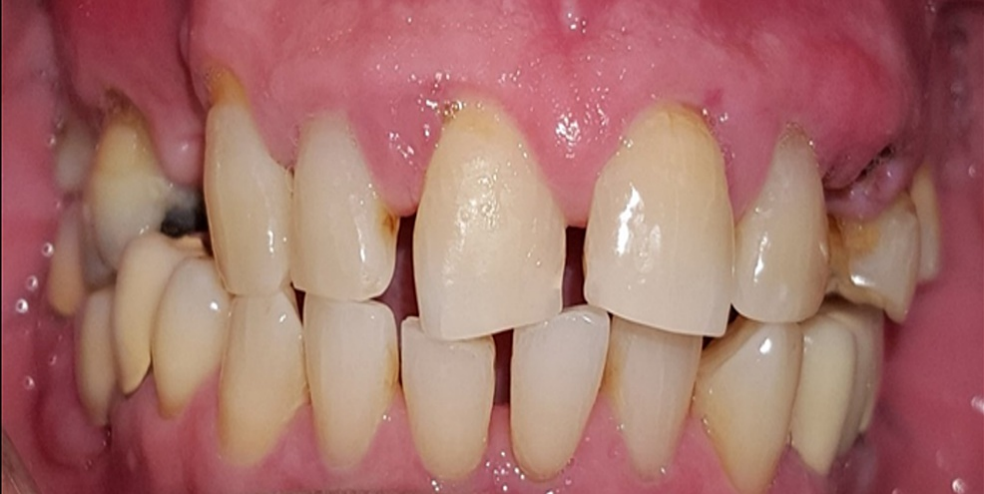
Presentation after non-surgical periodontal therapy and cessation of felodipine. Presence of parulis (gum boil) on the buccal aspect of attached gingiva in relation to the missing tooth 14.

**Figure 6:**
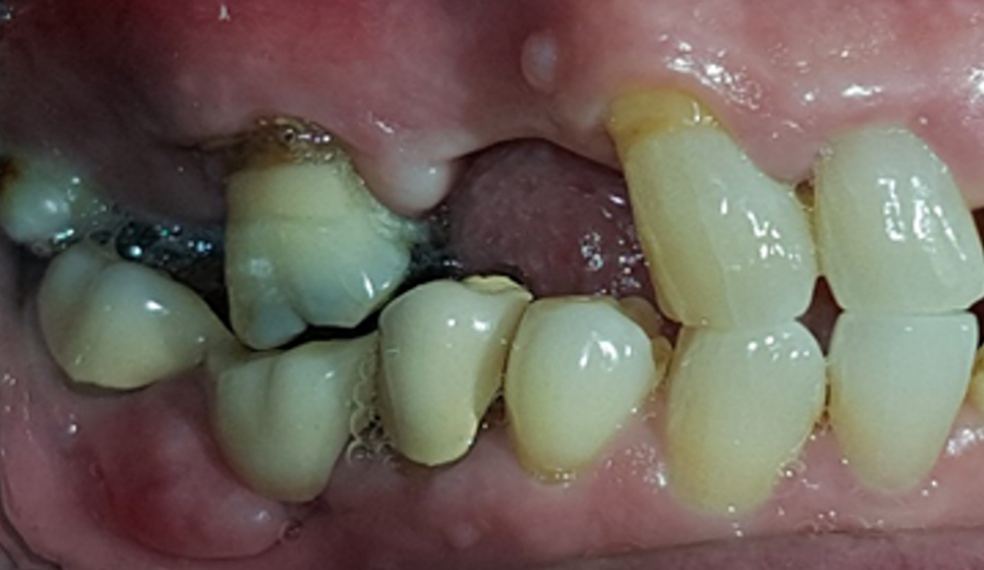
Persistent DIGE beneath the pontic of the bridge of teeth 45 to 47. Over erupted 16 compromised the prosthesis design. Presence of parulis (gum boil) on the buccal aspect of attached gingiva in relation to the missing tooth 14.

**Figure 7:**
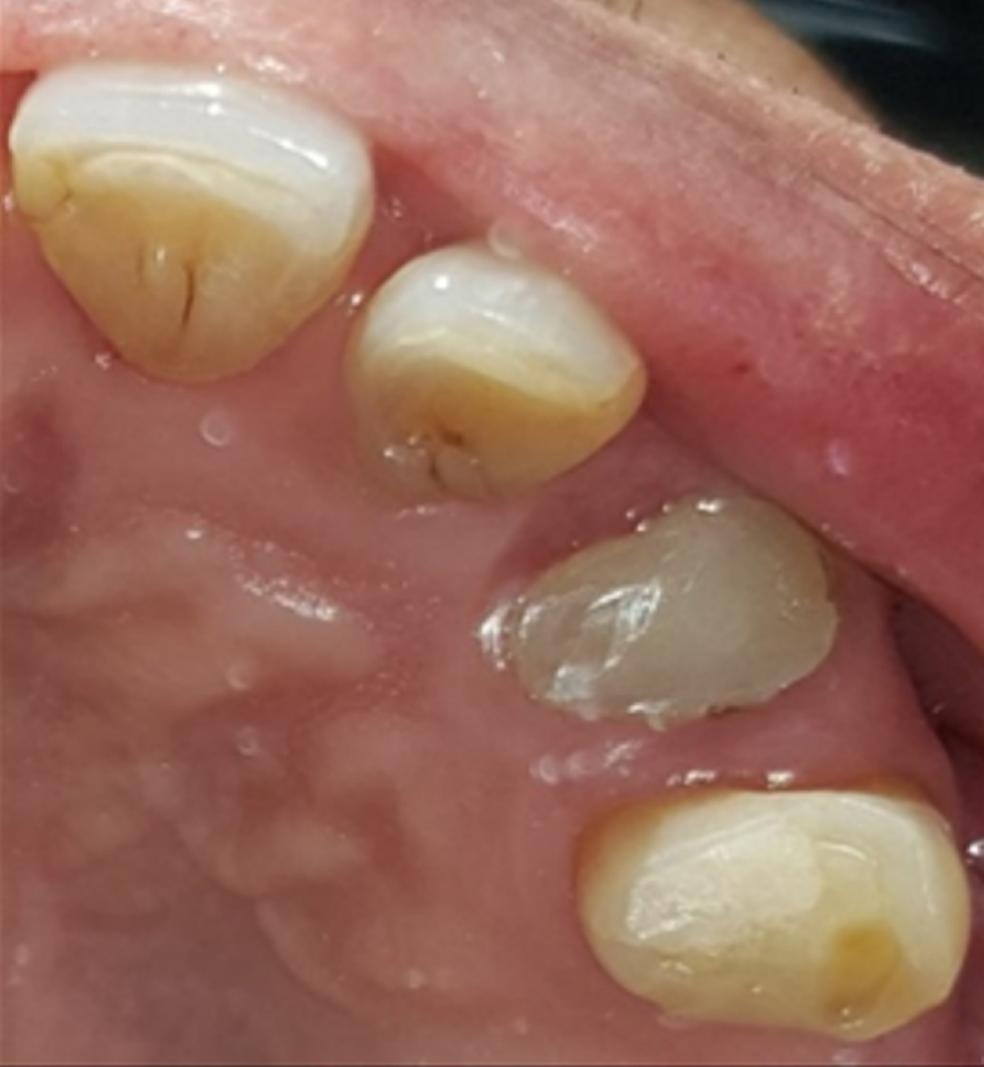
Root canal retreatment on retained root 23 and restored with direct composite.

**Figure 8:**
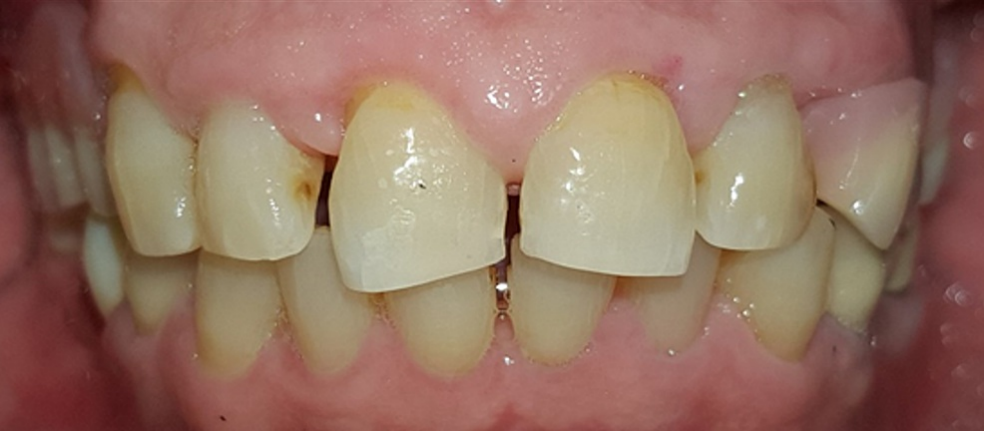
Upper interim acrylic RPD in-situ.

The management of this DIGE involved first non-surgical periodontal therapy (NSPT) and a review after 4 to 6 weeks. Oral hygiene instruction was given, including the use of super floss and mouthwash, followed by full mouth scaling and polishing. A 0.2% chlorhexidine mouthwash was prescribed for homecare. Upon five weeks review, the DIGE had not subsided; hence, a medical referral was made. The patient was previously prescribed with felodipine and valsartan for hypertension, but due to the DIGE issue, the medical team had advised the patient to cease the intake of felodipine and double the dosage of valsartan. One-month review later revealed the DIGE was resolved (Figure 5), except beneath the pontic of the poorly designed bridge of teeth 45 to 47 (Figure 6). Tooth 16 with a poor prognosis was planned for extraction, but the unstable patient’s blood pressure (BP) had prevented the treatment from being carried out. Meanwhile, root canal retreatment on tooth 23 was performed. The justification for this treatment was that tooth 23 has excellent periodontal support, except for the exposed coronal GP and absence of clinical ferrule; hence, endodontic retreatment has been commenced followed by composite restoration. Later, tooth 23 became a tooth-supported overdenture to preserve bone support (Fig. 7). Another medical referral was made to stabilize the patient’s BP. The team restarted the CCB using the third generation (Amlodipine 10mg OD) combined with beta-blockers (Metoprolol 30mg OD) in order to stabilize the patient’s BP. The patient had been warned about the risk of recurrent DIGE and was advised to liaise with both the medical and dental teams accordingly. Surprisingly, no recurrent DIGE was observed after three months, despite the fact that he was prescribed the same group of CCB but a different generation. Eventually, extraction of tooth 16 took place, and the patient was provided with an upper interim acrylic removable partial denture (RPD) to improve posterior support and mastication (Figure 8).

**Figure 9:**
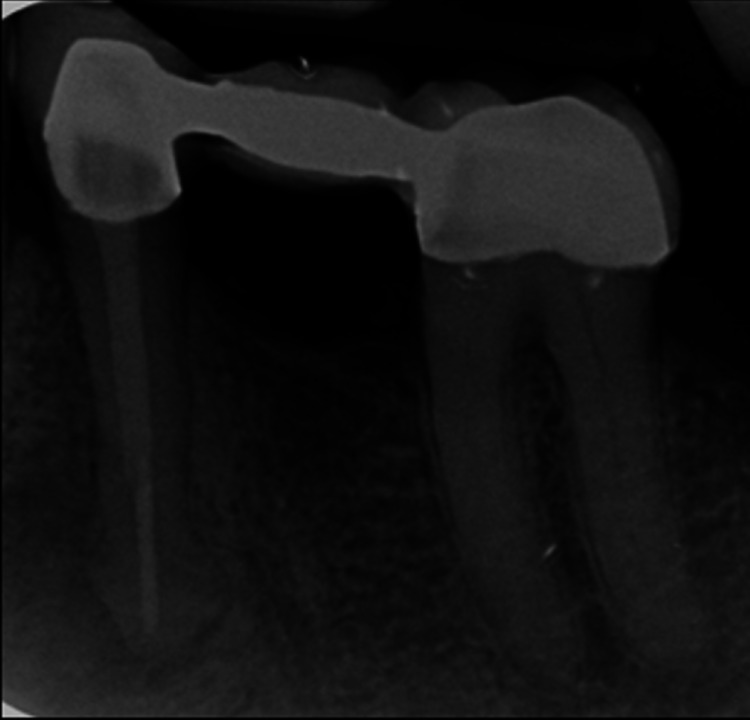
Radiograph of tooth 34 after the endodontic treatment and the new fixed-fixed conventional prosthesis. The intraoral periapical radiograph revealed a bulbous root tip in tooth 34, suggestive of hypercementosis.

**Figure 10:**
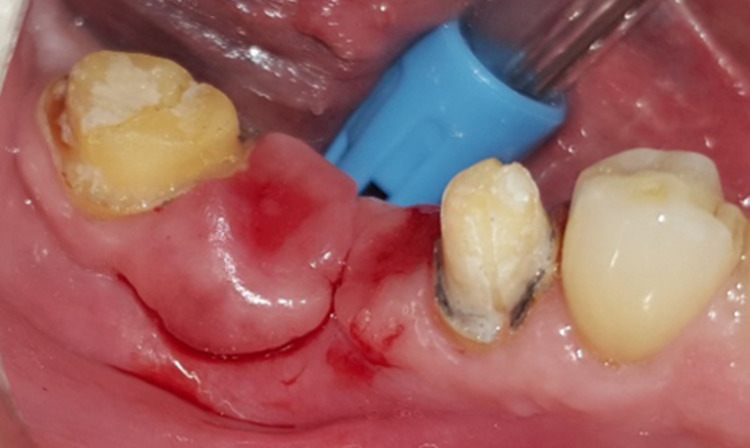
The appearance of the drug-influenced gingival enlargement fungating from the gingival margins of the abutments 45 and 47.

**Figure 11:**
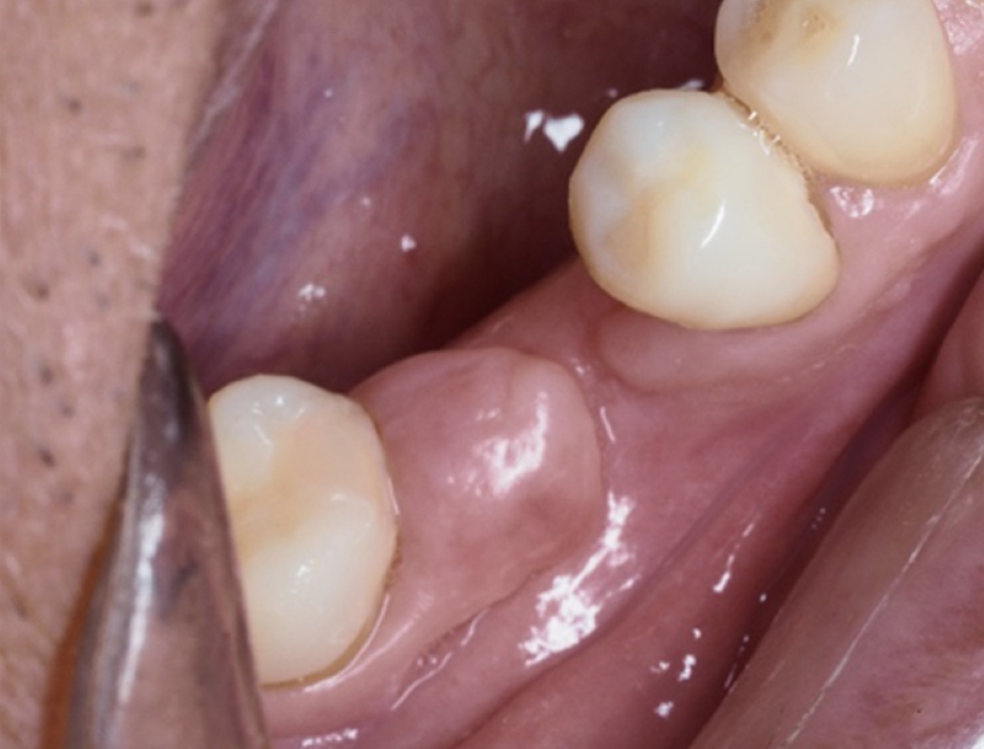
One-month postoperative revealed remaining DIGE. Later the lesion was subjected to gingivectomy.

**Figure 12:**
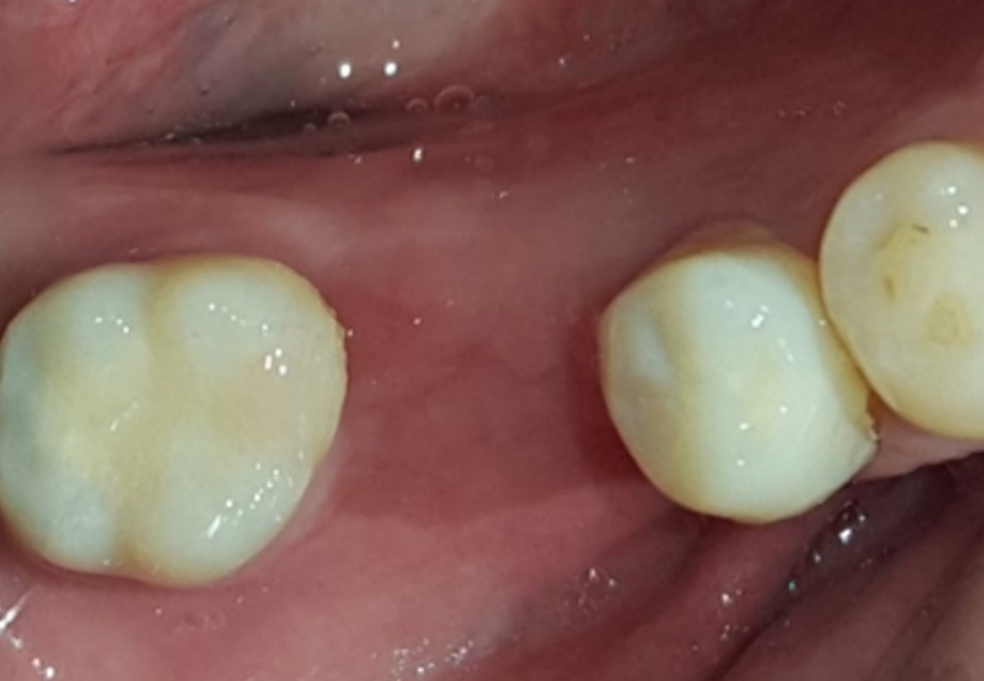
One-month postoperative gingivectomy revealed excellent soft tissue healing.

Root canal treatment was commenced on abutment 34 through the bridge retainer, followed by post and core placement prior to the new fixed-fixed conventional bridge (Figure 9). A poorly designed fixed-fixed conventional bridge of teeth 45 to 47 was removed (Figure 10), and the abutment teeth were evaluated for restorability and then restored with temporary crowns to allow soft tissue healing. The one-month review revealed the DIGE was dimensionally reduced but still not ideal to promote easy access for cleaning; hence, gingivectomy was done by excisional biopsy with a surgical blade (Figure 11). The tissue specimen was sent to the laboratory for histopathological examination, which confirmed the diagnosis of inflammatory fibro-epithelial hyperplasia. One-month post-gingivectomy revealed excellent soft tissue healing (Figure 12).

**Figure 13:**
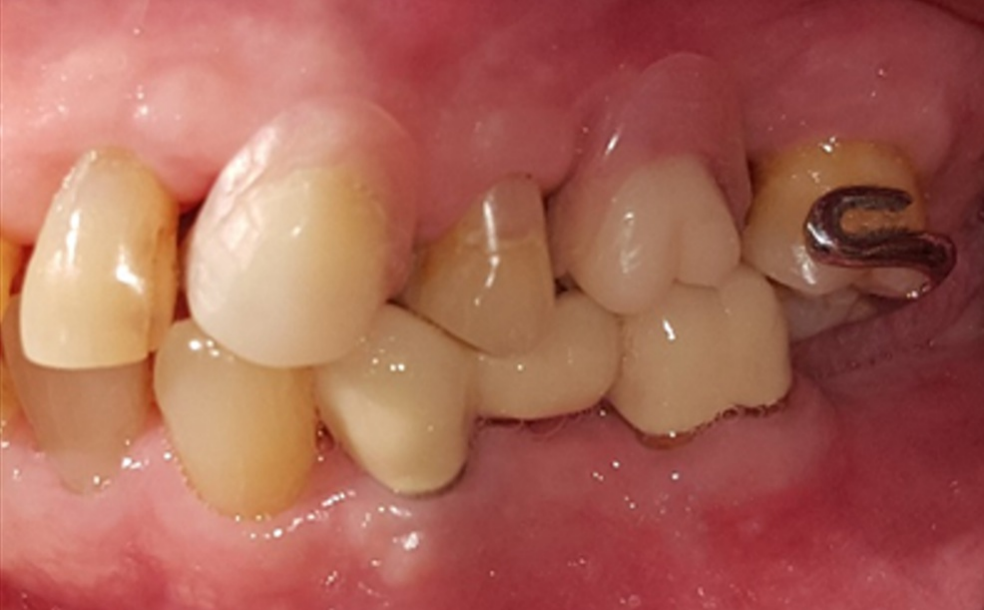
Three-unit fixed bridge of teeth 34 to 36 with hygienic pontic.

**Figure 14:**
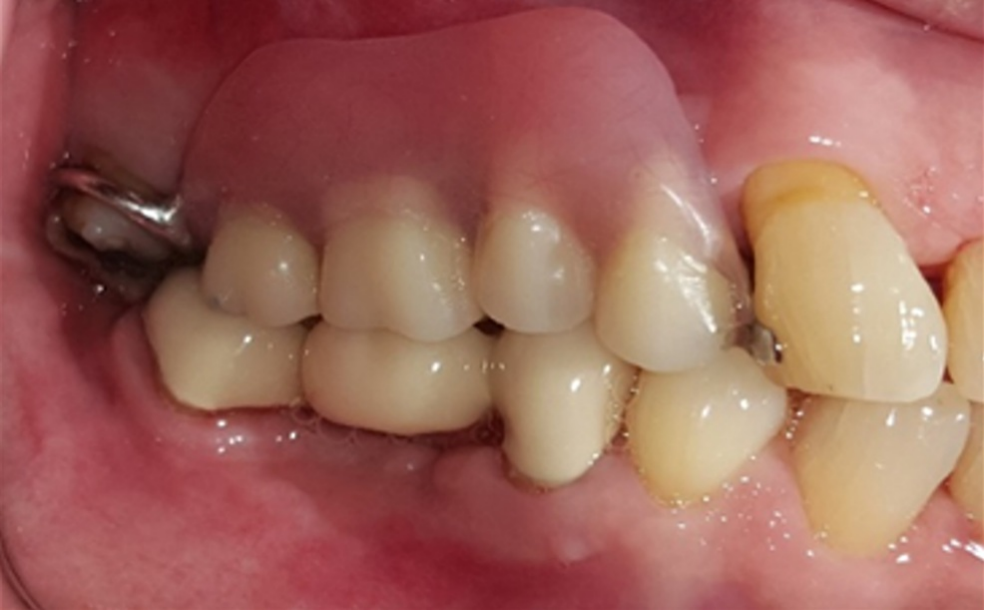
Modification of retentive clasp on tooth 13 and the new fixed prosthesis with hygienic pontic on teeth 45 to 47.

**Figure 15:**
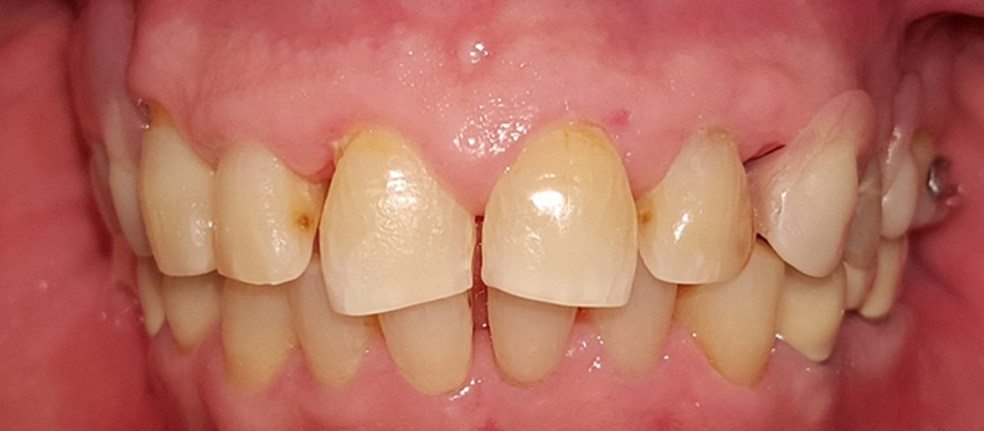
Post-operative clinical presentation upon review.

The bridges from 34 to 36 and 45 to 47 were redesigned with hygienic pontics (Figures 13, 14), allowing easier oral hygiene homecare for the patients. The interim upper acrylic RPD also has been replaced with a cobalt-chrome RPD. Since the patient has a gummy smile, it hinders the placement of the clasp on the anterior tooth. Modification of the retentive clasp on tooth 13 (Figure 14) has been proposed, allowing the provision of retention of the cobalt chrome RPD without compromising the aesthetic. The patient has been reviewed regularly (three monthly) to ensure pristine oral hygiene and monitor the risk of recurrent DIGE. The latest review indicated that there was no recurrent DIGE, and the patient was on a maintenance phase. The patient was pleased with the treatment provided (Figure 15).

## Discussion

The pathophysiology of DIGE remains elusive, as both inflammatory and non-inflammatory pathways may be involved in DIGE [7]. With regards to periodontal disease, it is recognized that plaque-induced gingival inflammation is a risk factor for DIGE, and several drug administrations will further complicate the cellular inflammatory events, particularly in the gingival tissue. The presence of a higher drug concentration in the serum will eventually lead to its detection in the gingival crevicular fluid, which may have an adverse effect on the existing inflammatory tissue. This may cause an upregulation of levels of several cytokines, including fibroblast growth factor 2, transforming growth factor-β1, interleukin-6 (IL-6), IL-1B, and platelet-derived growth-factor-β, which may lead to an increase in fibroblasts, contributing to DIGE [11]. As the cellular inflammatory events take place, there will be migration of mast cells and proliferation of fibroblasts, which eventually causes an increase in extracellular matrix synthesis and a decrease in degradation activity. Collectively, the administration of CCB has interfered with calcium ions homeostasis [6], which leads to a high intracellular calcium ions concentration, which at a later stage promotes the excessive influx of fibroblasts, leading to the pathophysiology of DIGE. The first clinical sign of gingival changes occurs within one to three months after the start of medication [12]. As for this patient, the GE developed after three months of taking felodipine. Due to the patient’s unstable blood pressure, the medical team restarted the CCB with amlodipine, which has been reported to have a low incidence of DIGE, 1.4% [13]. Surprisingly, there was no recurrent DIGE noted in this patient, which enabled the authors to conclude that the occurrence of DIGE was associated with biofilm-induced inflammatory DIGE but exacerbated by the felodipine, as stated in the final diagnosis.

The management of DIGE involved a thorough investigation of the patient’s background history, family members, medical health problems, medications taken, and oral hygiene practice status. As for this patient, both surgical and non-surgical approaches to periodontal therapy have been adopted to manage DIGE. The improvement of oral hygiene practice, the elimination of plaque retentive factors by non-surgical periodontal therapy, and the cessation of drug (felodipine) after a medical consultation proved to overcome the DIGE, except around the poorly designed fixed partial prothesis extending from teeth 45 to 47, which required surgical excision of the lesion [14]. As the periodontal issue has been resolved, oral rehabilitation continues to involve redesigning the fixed partial prostheses to facilitate the maintenance of periodontal health. The new fixed partial prostheses extending from teeth 34 to 36 and 45 to 47 were fabricated with hygienic pontics to prevent plaque accumulation as well as to provide easy access for cleaning, thereby helping patients to keep the prostheses clean compared to the previous bridges. There were a few recommended pontic designs for the mandibular posterior regions; hygienic pontic, modified ridge lap pontic, and conical pontic. Therefore, as the mandibular posterior teeth posed the least aesthetic concern, a conventional fixed-fixed conventional bridge with hygienic pontics was chosen in this case as it promotes cleansibility and pressure-free contact [15], hence avoiding plaque retention, which might cause the recurrence of DIGE. Tooth 16 was extracted due to poor prognosis based on these criteria: reduced periodontal support, presence of periapical disease and root caries, as well as the cause of occlusal derangement [16]. Root canal retreatment on tooth 23 was performed to serve as an abutment for the overdenture. It is crucial to preserve the bone around tooth 23 as part of the planning for implant placement in the future. A removable cobalt-chrome prosthesis was fabricated to improve the patient’s function of chewing food as well as to gain more posterior support, hence preventing heavy loading around the anterior teeth, which eventually may lead to localized tooth wear [17]. Another challenge encountered in treating this patient is the high smile line displayed, which can adversely affect the aesthetic outcome, especially tooth 13 as the abutment tooth for the removable prosthesis. A modification has been made to the retentive component of the cobalt chrome partial denture by taking into consideration the absence of the necessary undercut for the clasp to engage as well as the undesirable appearance of the retentive arm. Instead of the I-bar or C-clasp design, a ‘friction grip’ extension of the guide plane was adopted just below the survey line on the distal side, as shown in Figure 14.

## Conclusions

A comprehensive clinical assessment, together with an excellent rapport with the medical team, is critical in handling this DIGE case. Patients with DIGE must be monitored on a regular basis to detect any recurring growth, which may add to the cost, time, and energy spent on and by the patient. Effective communication and education between dentist and patient are crucial to ensure a holistic approach that benefits both parties.
